# Epidemiology of Tuberculosis in Kazakhstan: Data from National Electronic Healthcare System 2014-2019

**DOI:** 10.1093/eurpub/ckac131.124

**Published:** 2022-10-25

**Authors:** Y Sakko, M Madikenova, A Kim, A Gaipov

**Affiliations:** Department of Medicine, School of Medicine, Nazarbayev University, Nur-Sultan, Kazakhstan

## Abstract

**Background:**

Tuberculosis (TB) remains a global public health threat. WHO determined Kazakhstan as one of 18 high-priority countries for TB elimination in Europe, with reported TB incidence >20 cases per 100,000 population. There is a lack of comprehensive reseach of TB epidemiology in the country. This study aims to estimate the prevalence, incidence, mortality rates and survival hazard ratios of TB in Kazakhstan, using large-scale administrative health data records in 2014-2019.

**Methods:**

This is a population-wide retrospective study assessing 150 thousand TB (ICD10: A15-A19) patients’ incidence, prevalence and mortality. Demographic factors, diagnoses and comorbidities were analyzed. Univariate, bivariate and multivariate statistical analyses were performed. Cox regression and Kaplan-Meier survival analysis have been done.

**Results:**

Out of 150 thousand all TB patients, 61 percent were male and 94 percent had respiratory TB. During 2014-2019, the TB incidence, prevalence and mortality per 100K population declined (227-15.2), doubled (325.3-746.6) and increased (8.4-15.2), respectively. Age-specific TB incidence was lowest for 0-10 y.o., highest for 20 y.o. Being an old person, male, urban resident, retired, with HIV and diabetes was significantly associated with lower survival compared to a young, female, rural resident, employed, with no comorbidities.

**Conclusions:**

This was the largest TB study in Kazakhstan, presenting the country’s TB by demographic groups, incidence, prevalence, mortality trends, and risk factors against survival.

**Key messages:**

• During 2014-2019, the TB incidence, prevalence and mortality per 100K population declined (227-15.2), doubled (325.3-746.6) and increased (8.4-15.2), respectively.

• Being an old person, male, urban resident, retired, with HIV and diabetes was significantly associated with lower survival compared to a young, female, rural resident, employed, with no comorbidities.

